# Role of Magnetic Resonance Imaging in the Evaluation of Rotator Cuff Tears

**DOI:** 10.7759/cureus.21025

**Published:** 2022-01-08

**Authors:** Deepak V Koganti, Purnachandra Lamghare, Vinay Kumar Parripati, Rachit Khandelwal, Ayapaneni Dileep Reddy

**Affiliations:** 1 Radiology, Dr. D.Y. Patil Medical College, Hospital and Research Centre, Pune, IND

**Keywords:** supraspinatus, acromiohumeral distance, acromion, shoulder mri, rotator cuff tears

## Abstract

Background

Magnetic resonance imaging (MRI), with the advent of surface coils, is becoming the modality of choice for imaging soft tissues around the shoulder joint. Good knowledge regarding the MR characteristics of rotator cuff tendons, acromion, and the abnormalities in these tendons is necessary for appropriate diagnosis.

Methods

This was a hospital-based descriptive, analytical and prospective study conducted at our tertiary care hospital. The study was performed on 50 patients with rotator cuff lesions detected on MRI of the shoulder joint.

Results

The age distribution found in the study is between 19 and 66 years with mean being 43 ± 14.8 years. The peak incidence was found in the fifth and sixth decades of life. Gender-wise distribution of rotator cuff pathologies has shown no significant gender variation. The pain was the most common presenting complaint. An abnormal supraspinatus tendon was seen in 82% of the 50 study patients, making it the most commonly affected tendons, followed by subscapularis and infraspinatus tendons. No apparent teres minor pathology was identified in the study patients. The most common pathology affecting the supraspinatus tendon was tendinosis (38%) closely followed by a partial tear (36%). Among the partial tears, the articular surface type of tear was the most common. About 52% patients had type II (curved) acromion; making it the most common type of acromion followed by type III (hook), supraspinatus tendinopathy was more common in type II acromion. A reduction in the acromiohumeral distance can cause supraspinatus tendinosis and also makes it more susceptible to tear. About 45.5% showed supraspinatus tendon tears when the acromiohumeral distance was less than 8mm as compared to 13.6% when more than 10mm. Only 4.2% had normal supraspinatus tendon in patients with this distance less than 7mm.

Conclusion

MRI provides valuable information to the orthopaedic surgeon regarding the status of tendons, bones, and joints. In order to choose the appropriate course of action, it is crucial first to identify the issue and report relevant data from rotator cuff imaging. A full grasp of the rotator cuff's architecture and function, as well as the repercussions of rotator cuff diseases, is required.

## Introduction

The shoulder joint comprises the humerus, clavicle, and scapula, and it has a greater range of motion than any other joint while remaining stable in everyday settings. The shoulder complex comprises three joints: the glenohumeral, acromioclavicular, and sternoclavicular. The glenohumeral joint is a true synovial ball-and-socket style joint formed between the humeral head and the glenoid fossa of the scapula. It allows for a broad range of abduction, adduction, flexion, extension, internal/medial rotation, external/lateral rotation, and circumduction at the shoulder joint. It is the most mobile joint in the human body. It is also the least stable joint in the body and is the most dislocated diarthrodial joint. The acromioclavicular joint is a diarthrodial joint where the lateral clavicle articulates with the acromion process. Under normal physiological conditions, the acromioclavicular joint is a synovial joint that only glides. It promotes shoulder abduction and flexion by attaching the scapula to the thorax. It also enables force transmission from the upper arm to the skeleton [1]. The shoulder joint is multiaxial, allowing for a wide range of motion but sacrificing skeletal stability.

There are five stabilizers of the shoulder joint that are 1) rotator cuff muscles, 2) biceps tendon, 3) capsular ligaments, 4) glenoid labrum and 5) negative intra-articular pressure.1 and 2 are dynamic stabilizers, while 3-5 are static stabilizers. The rotator cuff muscles comprise supraspinatus, infraspinatus, subscapularis, and teres minor. 

This rotator cuff stabilizes and holds the humerus head in the glenoid cavity of the scapula. A musculotendinous collar surrounds the joint, providing stability except on the inferior aspect. The biceps brachii muscle tendon's long head passes superiorly through the joint, preventing the humeral head from sliding up into the glenoid cavity. The rotator cuff tendons are frequently affected by impingement and tendinopathy. It is a dynamic process that begins with degradation and culminates in tears in these tendons. There is an increasing recognition that rotator cuff illnesses are multifaceted, including exterior and internal processes - the extrinsic mechanism is based on microtrauma causing strain on the tendon, resulting in microtear [2]. According to the intrinsic mechanism, tendon degeneration and zones of critical vascularity predispose to tendon tear even in a low energy mechanism. Because the rotator cuff is one of the most important stabilizers of the shoulder joint, its injury can cause severe joint dysfunction, such as stiffness, restricted or painful joint movements, and even restricting daily activities. Conventional radiography, ultrasonography (USG), computed tomography, magnetic resonance imaging (MRI), and arthrography play an essential role in shoulder joint imaging and diagnosing rotator cuff pathologies. During rotator cuff imaging, it is also crucial to identify the condition, appreciate the potential clinical consequences, and report relevant results [3].

Conventional radiography and computed tomography (CT) give valuable information about the bones that comprise the joints, such as degenerative changes in the bones and joints, osteophytosis, and spur development. They also enable more precise measurements of the acromiohumeral distance (AHD) and coracohumeral interval, both shortened when the underlying tendons have impinged.

USG and MRI can evaluate the soft tissue structures around the shoulder joint, notably the cuff tendons. The results of the USG and MR examinations were nearly comparable [4]. The advantage of sonography is that it allows for dynamic real-time examination, but USG is very dependent on the operator. MRI has a high spatial resolution for examining soft tissue, such as tendon swelling and muscle cuff rips. When using MR, tear categorization becomes more visible. It also has the advantages of excellent multiplanar delineation without contrast and the absence of radiation hazards. MRI provides detailed information on cuff problems and surrounding structures, muscle atrophy, cross-sectional muscle area, and fatty degeneration, all of which have implications for the rotator cuff's physiologic and mechanical health [3]. Artefacts caused by respiratory and cardiac motions are not a problem in joint MRI, as they are in whole-body MRI [5]. The advantage of MR Arthrography is that it provides a more accurate delineation of ligament, labrum, and rotator cuff problems. It is, however, obtrusive and strongly reliant on the operator's knowledge. Currently, MRI, with the advent of surface coils, is becoming the modality of choice for imaging soft tissues around the shoulder joint. Good knowledge regarding the MR characteristics of rotator cuff tendons and the abnormalities in these tendons is necessary for appropriate diagnosis and planning of management.

Against this backdrop, the study's objectives will allow us to better understand the pathological conditions of the rotator cuff tendons and provide insight into the complexities involved in the MR imaging characteristics of the rotator cuff disorders predisposing factors. In addition, the gender and age distribution among the study group, the presenting symptoms, and the implications associated with rotator cuff pathologies, if any, will be examined.

## Materials and methods

Study Design

This study is a prospective, descriptive and analytical type of study. The study was performed on 50 patients with rotator cuff lesions detected on MRI of the shoulder joint at the Department of Radiodiagnosis, Dr. D.Y. Patil Hospital, Medical College and Research Centre, Pune, during the study period between July 2019 and September 2021. It was to describe rotator cuff pathologies' MRI features and examine the influence of age and sex in the distribution of rotator cuff pathology among the population studied. The MRI was done on the advice of the referring doctor, and no patient was made to undergo MRI for the sole purpose of this study. All the MRI studies were performed on Siemens Vida Magnetic Resonance Imaging (3 Tesla). Institutional Ethics Sub-Committee (IESC), Dr. DY Patil Hospital, Medical College and Research Centre, Pune clearance was obtained before the start of the study. The research approval number is IESC/PGS/2019/167.

Inclusion criteria

Patients of age more than 15 years were included. All patients clinically suspected of Rotator cuff pathology were included in this age group.

Exclusion criteria

Postoperative cases with orthopaedic hardware were excluded. Patients with claustrophobia, cardiac pacemakers, metallic foreign body, bio stimulators, neurostimulators, and cochlear implants in-situ were excluded.

Data collection 

The patients identified based on inclusion and exclusion criteria were subjected to MR evaluation. The Proforma was designed based on the objective of the study. Necessary clinical history, physical and systemic examination findings were noted. In addition, patients were subjected to a radiograph of the shoulder joint anteroposterior view (AP) or high-resolution USG as and when required. On T1- and T2-weighted images, a full-thickness rotator cuff tear will be defined as a focal, well-defined area of increased signal intensity spanning the entire tendon, from the bursa to the articular surface. In contrast, partial-thickness rotator cuff tears will be considered when the fluid signals do not traverse the entire thickness of the tendon, as in the case of a partial-thickness tear.

Statistical analysis

Statistical analysis was performed by entering the data into Microsoft Excel and evaluating it using the SPSS Statistics software version 17.0 (IBM, Armonk, NY). The mean and standard deviation of age were calculated (SD). The gender of patients and their presenting complaints were reported in percentages. Supraspinatus, infraspinatus, and subscapularis tendon pathologies were classified as normal, tendinopathy, partial, and complete tears, and percentages were calculated. Additionally, the percentages of acromion types and AC joint configurations were reported. The chi-squared test was used to determine the association between AHD, acromion type, and supraspinatus tendon pathologies. The level of statistical significance was set at 0.05.

## Results

Fifty cases of rotator cuff disease patients were included in the present study. The study participants' mean (SD) age was 43 ± 14.8 years, with a minimum of 18 and a maximum of 66 years. A maximum percentage (22.0%) of rotator cuff pathology has been observed in the age group of 51-60 years. More than half (56%) of our patients were males, and the remaining 44% were females. The most frequent complaint was pain alone which was seen in 20 patients (40%). In descending order of frequency, other complaints are stiffness of joint among 10 (20%), a combination of pain and stiffness among 9 (18%), difficulty in raising the arm among 7(14%), weakness among 4(8%). Other less common symptoms associated with pain were numbness in two patients and overlying skin discolouration in one patient.

Supraspinatus tendon pathology

**Figure 1:**
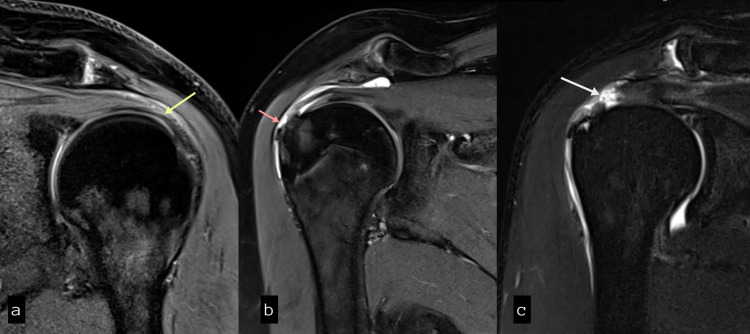
Coronal proton density fat saturation (PDFS) images showing (a) tendinopathy, (b) partial thickness tear, and (c) complete thickness tear of the supraspinatus tendon.

In the supraspinatus tendon, nine (18%) were average, and the remaining 41 (82%) had one of the pathologies. Out of those 41 patients who had pathology, 19 (38.0) had tendinopathy, 18 (36%) had a partial tear, and the remaining 4 (8.0%) had a complete tear (Figures 1a-1c, Table 1).

**Table 1:** Supraspinatus tendon pathology

Supraspinatus tendon pathology	Number	Percentage
Normal	9	18.0
Tendinopathy	19	38.0
Partial tear	18	36.0
Complete tear	4	8.0
Total	50	100.0

**Figure 2:**
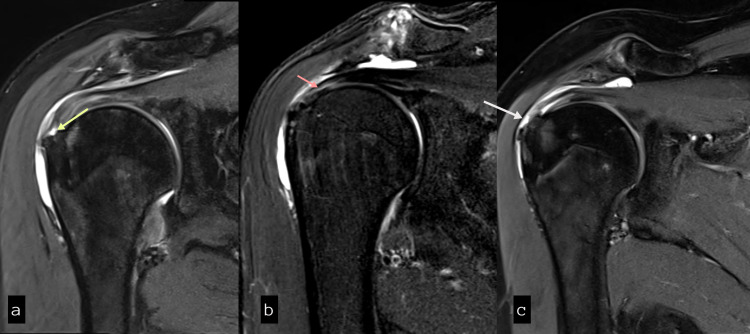
Coronal proton density fat saturation (PDFS) images showing (a) articular surface, (b) intra-substance, and (c) bursal surface types of partial-thickness tears of the supraspinatus tendon.

Partial tears can be further classified as intra-substance tear when it does not involve the surfaces, articular surface partial tear when the humeral side of the supraspinatus tendon is involved, and bursal surface partial tear when the acromial side is involved. Out of all the patients who had partial tears, nine (50%) had an articular tear, three (16.7%) had substance tear, and the remaining six (33.3%) had a bursal tear (Figures 2a-2c).

Infraspinatus and subscapularis tendon pathology

In the infraspinatus tendon (Table 2), out of the nine patients who had pathology, four (8.0%) had tendinopathy, four (8%) had a partial tear and the remaining one (2.0%) had a complete tear. In the subscapularis tendon (Table 3), out of the 21 patients who had pathology, 15 (30.0%) had tendinopathy, five (10%) had a partial tear and the remaining one (2.0%) had a complete tear.

**Table 2:** Infraspinatus tendon pathology

Infraspinatus tendon pathology	Number	Percentage
Normal	41	82.0
Tendinopathy	4	8.0
Partial tear	4	8.0
Complete tear	1	2.0
Total	50	100.0

**Table 3:** Subscapularis tendon pathology

Subscapularis tendon	Number	Percentage
Normal	29	58.0
Tendinopathy	15	30.0
Partial tear	5	10.0
Complete tear	1	2.0
Total	50	100.0

Type of acromion

**Figure 3:**
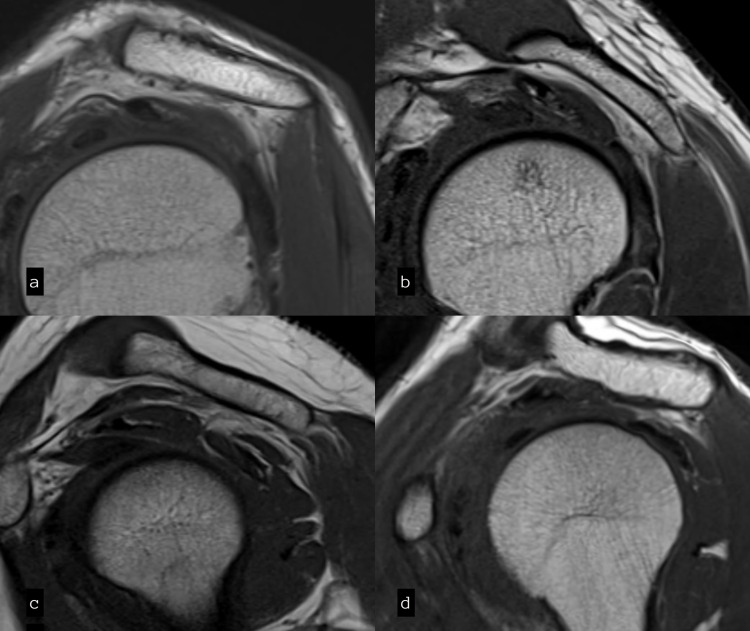
Sagittal T1 images showing acromion types: (a) Flat – Type I, (b) Curve – Type II, (c) Hook – Type III, and (d) Convex – Type IV.

A flat type of acromion was seen in seven (14%) of the patients, and more than 50% had a curved type of acromion (Figures 3a-3d). The remaining 34% of patients had a hook and convex type of acromion. In this study, the curve type of acromion is commonly seen (Table 4). In this study, 17 (34%) of the participants had a horizontal acromion, while 12 had an inferolateral tilt of the acromion (24%). Two people (4%) had acromion with low placement. Two (16.7%) of the 12 patients with inferolateral tilt had normal supraspinatus tendons, three (25%) had tendinosis, and five (41.6%) had partial tears, and two (16.7%) had complete tears. Thirteen patients (26%) had posterior downsloping, and six (12%) had anterior downsloping acromion. Three (17.6%) of the 17 patients with horizontal or upward acromion had normal tendons, four (23.5%) had tendinosis, and 10 (58.8%) had tears (Table 5).

**Table 4:** Type of acromion

Type of acromion	Number	Percentage
Flat (Type I)	7	14.0
Curve (Type II)	26	52.0
Hook (Type III)	14	28.0
Convex (Type IV)	3	6.0
Total	50	100.0

**Table 5:** Acromioclavicular joint configuration

Acromioclavicular configuration	Number	Percentage
Horizontal	17	34.0
Posterior down slopping	13	26..0
Anterior down slopping	6	12.0
Inferior lateral tilt	12	24.0
Low placed	2	4.0
Total	50	100.0

Association between AHD and supraspinatus tendon

The AHD is measured between the humeral head's superior articular surface and the acromion's under-surface. The patients were grouped into less than 8mm, 8-10mm, and more than 10mm. Tendinopathy was the most common and highest (54.2%) in patients with an AHD of less than 8mm and the least percentage (11.1%) observed in more than 10mm of AHD. The partial tear was high (41.2%) in 8-10 mm of distance, and complete tear was also high in the same group of patients. The association between the AHD and supraspinatus tendon pathology was statistically significant, with a p-value of 0.02 (Table 6).

**Table 6:** Association between acromiohumeral distance and supraspinatus tendon

Acromiohumeral distance	Supraspinatus tendon pathologies							
	Normal		Tendinopathy		Partial tear		Complete tear	
	n	%	n	%	n	%	n	%
<8mm	1	4.2	13	54.2	8	33.3	2	8.3
8-10mm	3	17.6	5	29.4	7	41.2	2	11.8
>10mm	5	55.6	1	11.1	3	33.3	0	-
Total	9	18.0	19	38.0	18	36.0	4	8.0

Association between supraspinatus pathologies and type of acromion

Supraspinatus partial tear was highest in the convex type of acromion 2 (66.7%) in this study (Table 7).

**Table 7:** Association between supraspinatus pathologies and type of acromion

Supraspinatus tendon pathologies	Types of acromion							
	Flat		Curve		Hook		Convex	
	n	%	n	%	n	%	n	%
Normal	1	14.3	2	7.7	5	35.7	1	33.3
Tendinopathy	3	42.9	13	50	3	21.4	0	-
Partial tear	3	42.9	8	30.8	5	35.7	2	66.7
Complete tear	0	-	3	11.5	1	7.1	0	-
Total	7	100	26	100	14	100	3	100

## Discussion

MRI has become a powerful tool for evaluating musculoskeletal disorders with the introduction of surface coils. MRI imaging of the shoulder has numerous advantages over traditional methods. Because the soft tissue components that support the shoulder are structured in numerous planes, MRI's direct multiplanar imaging capabilities are superior to computed tomography's single plane capability. MRI clearly shows the rotator cuff, and the individual core tendons of the four rotator cuff muscles can be distinguished [6]. As a result, rotator cuff anomalies can be precisely located and quantified. The rotator cuff can be seen clearly on MRI, including the subacromial section, which is not seen on USG. The rotator cuff muscles are responsible for stabilizing the shoulder joint and are frequently impacted.

Rotator cuff disease distribution by age

The patients' ages with rotator cuff problems in this study varied from 19 to 66 years, with a mean of 43.3 ± 14.8 years. The fifth and sixth decades of life were found to have the highest occurrence. According to several studies, the incidence of rotator cuff tendon degradation and injury increases with age. According to Ozaki et al. and Uhthoff, the pathogenesis of rotator cuff diseases is an intrinsic process [7-9]. Rotator cuff dysfunction is more common with increasing age. Microvascular investigations have revealed decreased vascularity in the cuff tissue as people get older, which matches the degeneration pattern seen in age-related degenerative tendinopathies [10-12]. In our study, a complete tear was noticed most common in above 50 years.

Rotator cuff illness gender distribution

The majority of the research describes an ambiguous distribution of rotator cuff illness. Out of the 50 patients with rotator cuff pathologies, 28 were male patients (52%), and 22 were female patients, indicating no significant difference in gender distribution among the study group; this is in line with Milgrom et al.'s research of 90 individuals, which found no statistically significant variations in the incidence of rotator-cuff lesions based on gender [13].

Rotator cuff disease and clinical complaints

The most common symptom linked with rotator cuff disease is pain. Pain is usually minor when the arm is in a neutral and supported position. When combing hair, the pain is usually aggravated by overhead rising or abduction of the arm [14]. The inability to elevate the arm above shoulder level is a common sign of actual weakness. The most common complaint of rotator cuff problems in our sample was pain, which is consistent with the literature [14].

Rotator cuff pathologies

Impingement causing tendinosis, partial and full-thickness rotator cuff tears, calcific tendinitis, and coracoid impingement affecting the subscapularis tendon are all rotator cuff illnesses. The pathomorphological alterations in the rotator cuff are likely complex, with both intrinsic and extrinsic variables playing a role. This study looks at characteristics such as acromion type, and acromial orientation. The supraspinatus tendon was the most afflicted in our study, followed by the subscapularis and infraspinatus. Teres minor was not found in any of the study's patients, which is in line with the findings of Jerosch et al.'s investigation [15]. However, it was discovered in a study of dissected shoulder joint specimens from 122 people. In 78% of cases, isolated supraspinatus was found [15].

In a study of cadaver shoulders, De Palma et al. found that supraspinatus injury was the most common, and its severity increased with age [16]. On short TR/TE (e.g.) T1 weighted image and proton density images, tendinosis or tendinopathies were identified by a relatively highly intense signal within the tendon. The signal intensities are aligned along the tendon's long axis. They can be inhomogeneous (focal, diffuse, or bandlike) or homogeneous (focal, diffuse, or bandlike). Partially torn tendons can occur intratendinously/intra-substance, without contacting the surface, on either the bursal or articular surface of the tendon. On both short and long TE sequences, partial rips emerge as discrete regions of hyperintensity on MRI. The hyperintense signal does not extend the complete length of the tendon. Partial tears were defined as defects or discontinuities in the tendon fibres that did not extend through the tendon's thickness in our study of 50 patients with shoulder complaints. Hyperintense signal in T1 weighted images with corresponding T2 hyperintensity, with defects or discontinuity in the tendon fibres that do not involve the full thickness of the tendon, were termed as partial tears. In total, 27 of the 50 patients had partial tears (54%): 18 (36%) in the supraspinatus tendon, four (8%) in the infraspinatus tendon, and five (10%) in the subscapularis tendons.

Fluid in the subdeltoid bursa is frequently detected in bursal-side partial-thickness tears, making it easier to quantify the extent and depth of these tears. Surface rips of the articular cartilage are more prevalent than bursal surface tears. Unfortunately, this could also make the tendon susceptible to articular surface partial-thickness tears. On at least one image, full-thickness tears occur when signal anomalies appear to stretch over the entire thickness, spanning from surface to surface. The presence of fluid resulting from a tear causes this strong signal strength. Complete or full-thickness tears are more common in supraspinatus tendons than other rotator cuff tendons. Full-thickness tears were detected in six out of 50 patients (12%) in this investigation, with four (8%) occurring in the supraspinatus tendon, which is consistent with the literature.

Teres minor is a critical external rotator of the shoulder, accounting for up to 45% of external rotation power. Whether intact or hypertrophied, the teres minor can supply adequate force to external rotation and maintain the capacity to do daily activities like eating and drinking while also reducing the symptoms of the other cuff tendon tear. From September 1996 to April 2002, a study was done on 2,436 shoulders MRI tests over 67 months [17]. Only 0.8% of the study population had an MRI finding of teres minor abnormalities. In our analysis, none of the teres minors showed any abnormalities. According to Bigliani and colleagues [18], acromion is categorized into four types: a flat, curved inferior surface, hooked and convex near the distal end. There is currently no statistically significant link between type IV and impingement in the literature. However, shoulder impingement is frequently related to types II and III.

In our study, a flat type of acromion was seen in seven (14%) of the patients, and more than half the patients (52%) had a curved type of acromion. The remaining 34% of patients had a hook and convex type of acromion. In this study, the curve type of acromion is commonly seen. Supraspinatus tendinosis and tears occurred more frequently in type II of the acromion. As a result, aberrant tendons were prevalent with type II/III acromion in this investigation. In a study of 140 cadavers, Biglialni et al. discovered a significant association between full-thickness rotator cuff tendon tears with type II acromion [18]. Ellman, in his research study, has described a similar association [19].

The space available for the supraspinatus tendon is greatly affected by acromion shape and slope, notably during abduction and rotational movements. The acromion's slope is best seen in sagittal and coronal oblique images. On coronal oblique views, the inferior cortex of the acromion should be aligned with the inferior cortex of the clavicle. If the inferior acromion cortex is exposed, it forms a low-lying acromion positioned under the clavicle's inferior cortex [20]. The acromiohumeral space narrows due to this configuration, which can lead to impingement. In this study, 17 (34%) of the participants had a horizontal acromion, while 12 had an inferolateral tilt of the acromion (24%). In addition, two people (4%) had acromion with low placement. Two (16.7%) of the 12 patients with inferolateral tilt had normal supraspinatus tendons, three (25%) had tendinosis, and five (41.6%) had partial tears, and two (16.7%) had complete tears. Thirteen patients (26%) had posterior downsloping, and six (12%) had anterior downsloping acromion. Three (17.6%) of the 17 patients with horizontal or upward acromion had normal tendons, four (23.5%) had tendinosis, and 10 (58.8%) had tears. Acromioclavicular joint abnormalities increased with age in research by Needell et al. in 100 participants [21].

The primary limitations of our study are the small sample size and the lack of correlation with arthroscopic findings. A large sample study with more intricate clinical, pathological, and surgical correlation would provide more details regarding the disease progression and help develop a protocol for patient management and draw broader inferences and enhance the current study's findings. Therefore, more research with a more extended study period is recommended.

## Conclusions

MRI has an invaluable role in diagnosis, grading, and treatment planning of rotator cuff injuries; this fact is further augmented by the results of this study. We would propose its use as a first-line imaging modality in all suspected cases of rotator cuff tears. When deciding on treatment, MRI provides valuable information to the orthopaedic surgeon regarding the status of tendons, bones, and joints. A full grasp of the rotator cuff's architecture and function, as well as the repercussions of rotator cuff diseases, is also required.
